# High prevalence and excess mortality of late presenters among HIV-1, HIV-2 and HIV-1/2 dually infected patients in Guinea-Bissau - a cohort study from West Africa

**DOI:** 10.11604/pamj.2016.25.40.8329

**Published:** 2016-09-29

**Authors:** Bo Langhoff Hønge, Sanne Jespersen, Johanna Aunsborg, Delfim Vicente Mendes, Candida Medina, David da Silva Té, Alex Lund Laursen, Christian Erikstrup, Christian Wejse, The Bissau HIV cohort study group

**Affiliations:** 1 Bandim Health Project, Indepth Network, Bissau, Guinea-Bissau; 2 Department of Infectious Diseases, Aarhus University Hospital, Denmark; 3 Department of Clinical Immunology, Aarhus University Hospital, Denmark; 4 National HIV Programme, Ministry of Health, Bissau, Guinea-Bissau; 5 GloHAU, Center for Global Health, School of Public Health, Aarhus University, Denmark

**Keywords:** HIV, late presenters, advanced disease, Guinea-Bissau, HIV-2

## Abstract

**Introduction:**

HIV infected individuals with late presentation (LP) and advanced disease (AD) have been associated with higher mortality, higher cost of medical management, impaired CD4 cell count increment and potentially ongoing risk of HIV transmission. Here we describe the proportion of patients with LP and AD at an HIV clinic in Guinea-Bissau, identify risk factors and evaluate the outcome of these patients.

**Methods:**

We included all patients >15 years diagnosed with HIV-1 and/or HIV-2 at the outpatient HIV clinic at Hospital National Simão Mendes, during June 2005 - December 2013 in a retrospective cohort study. Patients were followed until December 2014. LP and AD was defined as a baseline CD4 cell count of 200-349 cells/µL and <200 cells/µL, respectively.

**Results:**

A total of 3,720/5,562 (65.7%) patients had a CD4 cell count measured within the first 90 days of HIV diagnosis. Forty-eight percent had AD and 23% had LP. Risk factors for presentation with AD were male sex, age >30 years, Fula and Mandinga ethnicity. HIV-2 and HIV-1/2 dually infected patients had lower risk of AD compared with HIV-1 infected patients. Although antiretroviral therapy (ART) was initiated for 64.4% of patients, those with AD progression had a 3.82 times higher mortality compared to patients with non-LP.

**Conclusion:**

The majority of HIV infected patients presented late. Most of the late-presenters had advanced disease and patients with advanced disease had a very high mortality. Initiatives to enroll patients in care at an earlier point are needed and should focus on risk groups.

## Introduction

The introduction of antiretroviral treatment (ART) for HIV infection has significantly reduced the morbidity and mortality of HIV infected individuals [1]. However, the success of treatment depends on the disease progression at HIV diagnosis and adequate initiation of ART [2, 3]. Low CD4 cell count at HIV diagnosis termed late presentation (LP) and late presentation with advanced disease (AD) have been associated with higher mortality [4], higher cost of medical management [5], impaired CD4 cell count increment [6, 7] and potentially ongoing risk of HIV transmission [8, 9]. Furthermore, lag time between CD4 cell count measurement and subsequent medical consultation where ART initiation is decided, may contribute to further disease progression and loss to follow-up [10, 11]. Thus, intensified efforts are needed to identify and enroll HIV infected individuals earlier into care [12]. In West Africa, two types of HIV are circulating: HIV-1 and HIV-2. The UNAIDS estimates that in this part of the world less than half of the HIV infected individuals in need of ART are actually receiving the treatment [1]. The West African country Guinea-Bissau has the world´s highest prevalence of HIV-2 and the prevalence of HIV-1 has been rising [13]. Compared with HIV-1, HIV-2 is less transmissible, associated with a lower viral load and with a slower rate of both CD4 cell decline and clinical progression. Still, it may lead to AIDS, with clinical features indistinguishable from the syndrome caused by HIV-1 [14–16]. In this study, we describe the proportion of HIV-1 and/or HIV-2 infected patients with LP and AD at an HIV clinic in Bissau, to identify risk factors thereof and to evaluate the outcome of these patients.

## Methods

### Setting and patients

In June 2005, the first HIV clinic opened in Bissau, the capital of Guinea-Bissau, providing ART free-of-charge, and the clinic is now the largest ART center in the country in terms of patients on follow-up. CD4 cell count measurements have been performed since 2007, and the same year the Bissau HIV Cohort was established to evaluate patient treatment and follow-up [**17**]. Routines at the clinic has previously been described [**18**]. In this retrospective cohort study, patients >15 years were enrolled at the HIV clinic at Hospital National Simão Mendes (HNSM) in Bissau, Guinea-Bissau in the period June 2005 - December 2013. Follow-up continued until December 2014. Active follow-up of absent patients were performed at regular intervals by calling patients or their contact person(s) by telephone. Patients were considered lost to follow-up if on ART and absent for 90 days or without ART and absent for 210 days.

### Laboratory methods

CD4 cell counts were performed since April 2007 by flow cytometry using Partec CyFlow^®^ SL_3 (Cyflow SL, Partec, Munster, Germany) at the National Public Health Laboratory. HIV screening was done with a rapid test in the clinic (Determine HIV-1/2 assay, Abbott Laboratories, USA) and confirmation and discrimination using SD Bioline HIV 1/2 3.0 (Standard Diagnostics Inc, Kyonggi-do, South Korea). Since June 2012, the rapid test First Response HIV Card 1-2.0 (PMC Medical, Mumbai, India) has also been used for HIV type discrimination.

### Definition of LP and AD

Based on a consensus definition [**17**] we defined LP as patients presenting for care with a CD4 cell count below 350 cells/µL. AD were defined as patients presenting with a CD4 cell count below 200 cells/µL or presenting with an AIDS-defining event, regardless of the CD4 cell count. Only patients with a CD4 cell count measured within 90 days of HIV diagnosis were included in the analyses.

### Statistical methods

We compared the demographic, clinical and laboratory features of patients with AD, LP and non-LP using χ^2^ test for categorical variables. Logistic regression was used for the analysis of risk factors for LP and AD patients when comparing with non-late presenters. In case of missing data, a missing data (unknown) group was made and included in the analysis to avoid exclusion of patients. Variables associated with AD or LP in the univariable model (p<0.10) were included in a multivariable model unless the association was to the group of patients with missing data. Median CD4 cell count and median time to ART were compared between groups using the ANOVA test. Mortality rate ratios (MRR) were calculated using poisson regression analyses. All statistical analyses were carried out using Stata IC 13.0 (StataCorp, College Station, Texas, USA).

### Ethical statement

The Bissau HIV cohort has been approved by the national ethics committee in Guinea-Bissau (Parecer NCP/No.15/2007). Upon inclusion, the patients provided a voluntary, signed and dated informed consent, or fingerprint if illiterate.

## Results

### General characteristics

A total of 5,562 patients were diagnosed with HIV in the study period, but 68 (1.2%) patients were excluded as they were already receiving ART at first CD4 cell count. Furthermore, 1,843 patients (33.1%) were excluded from the analysis as CD4 cell count was not measured within the first 90 days after HIV diagnosis. Excluded patients were more likely to be single versus being married with an odds ratio (OR) of 1.36 (p < 0.01). In addition, a higher proportion of male patients did not have a CD4 cell count measured (OR 1.23, p < 0.01). In the subsequent analysis (Table 1), 3,720 HIV infected patients with a CD4 cell count measurement at diagnosis were included (69.6% HIV-1, 17.9% HIV-2, 10.4% HIV-1/2 and 2.1% HIV type unknown). A total of 2,478 (66.6%) of the patients were of female sex and the median age of all patients were 36 years (interquartile range, IQR 29-45 years).

**Table 1:** Characteristics of all included patients

	Advanced disease (AD)	Late presentation (LP)	Non-late presentation (non-LP)	
	n (%)	n (%)	n (%)	p-value
In total	1810	858	1051	
**HIV-type**				<0.01
HIV-1	1329 (73.4)	608 (70.9)	650 (61.8)	
HIV-2	262 (14.5)	144 (16.8)	260 (24.7)	
HIV-1/2	185 (10.2)	94 (11.0)	110 (10.5)	
Unknown	34 (1.9)	12 (1.4)	31 (3.0)	
**Sex**				<0.01
Female	1134 (62.7)	571 (66.6)	773 (73.5)	
Male	676 (37.3)	287 (33.4)	278 (26.5)	
**Age stratified**				<0.01
Age ≤ 30 years	466 (25.8)	246 (28.7)	339 (32.2)	
Age 30-49 years	1112 (61.4)	491 (57.2)	557 (53.0)	
Age ≥ 50 years	221 (12.2)	117 (13.6)	152 (14.5)	
Unknown	11 (0.6)	4 (0.5)	3 (0.3)	
**Marital status**				<0.01
Married	1014 (56.0)	443 (51.6)	576 (54.8)	
Divorced	118 (6.5)	36 (4.2)	67 (6.4)	
Widowed	235 (13.0)	131 (15.3)	169 (16.1)	
Single	414 (22.9)	236 (27.5)	221 (21.0)	
Unknown	29 (1.6)	12 (1.4)	18 (1.7)	
**Ethnicity**				<0.01
Balanta	268 (14.8)	168 (19.6)	209 (19.9)	
Fula	363 (20.1)	152 (17.7)	192 (18.3)	
Mandinga	189 (10.4)	78 (9.1)	72 (6.9)	
Manjaco	130 (7.2)	67 (7.8)	84 (8.0)	
Pepel	140 (7.7)	60 (7.0)	84 (8.0)	
Mancanha	95 (5.3)	68 (7.9)	70 (6.7)	
Other^+^	279 (15.4)	153 (17.8)	168 (16.0)	
Unknown	346 (19.1)	112 (13.1)	172 (16.4)	
**Attended school^++^**				0.42
Yes	1129 (62.4)	546 (63.4)	650 (61.9)	
No	609 (33.7)	287 (33.5)	370 (35.2)	
Unknown	72 (4.0)	25 (2.9)	31 (2.9)	
**Year of diagnosis**				0.01
2007	126 (7.0)	42 (4.9)	46 (4.4)	
2008	272 (15.0)	120 (14.0)	128 (12.2)	
2009	348 (19.2)	151 (17.6)	186 (17.7)	
2010	366 (20.2)	201 (23.4)	238 (22.7)	
2011	283 (15.6)	162 (18.9)	207 (19.7)	
2012	224 (12.4)	102 (11.9)	136 (12.9)	
2013	191 (10.6)	80 (9.3)	110 (10.5)	

+ Other ethnicities: Beafada, Bijago, Caboverdeano, Felupe, Mansonca and mixed

++ Not including koranic school

### Description of CD4 cell counts

**Figure 1:**
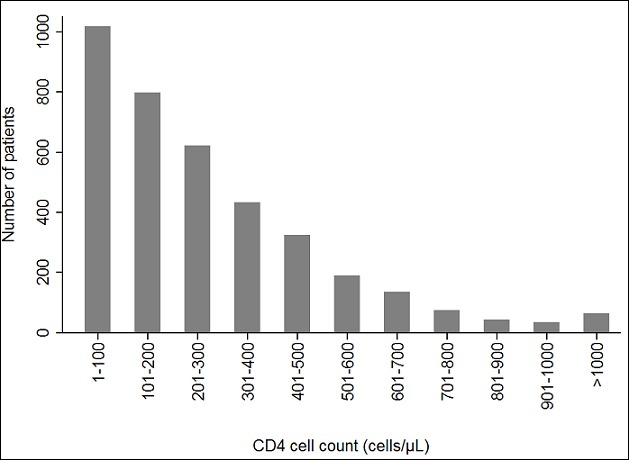
Number of patients by CD4 cell count at HIV diagnosis

**Figure 2:**
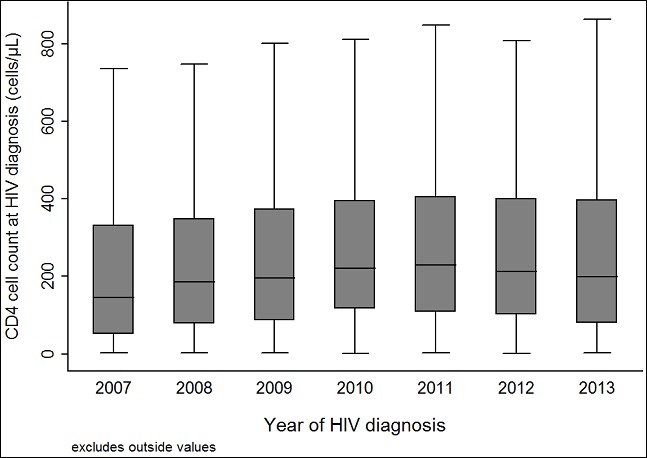
CD4 cell count by year of HIV diagnosis

The median time between HIV diagnosis and initial CD4 cell count measurement was 1 day (IQR 1-5 days). Only a smaller proportion (18.3%) of the patients had CD4 cell count measured the same day as the HIV diagnosis, whereas 33.9% had the analysis performed the following day. Figure 1 presents the CD4 cell count at HIV diagnosis of all patients included. Overall, the median CD4 cell count was 206 cells/µL (IQR 89 - 381). According to our definition, 1,810 (48.7%) patients had AD and 858 (23.1%) had LP; in total 71.8%. The median CD4 cell count at presentation varied during the study period (Figure 2) with the lowest measurements in 2007 (146 cells/µL) and the highest in 2011 (229 cells/µL, p<0.01). There was no significant difference between median CD4 cell count in 2011 and 2013 (199 cells/µL, p=0.33).

### Risk factors of LP and AD

Risk factors of AD in the multivariable analysis (Table 2) were male gender (adjusted odds ratio (aOR) 1.49), age 30-49 years (aOR 1.66), age =50 years (aOR 1.48), being single versus married (aOR 1.30), Fula ethnicity (aOR 1.47) and Mandinga ethnicity (aOR 2.04). The proportion of AD among patients with HIV-1 infection was 51.4%, HIV-2 infection 39.3% and HIV-1/2 dual infection 47.4%. Thus, when compared with HIV-1 the aOR of AD for HIV-2 and HIV-1/2 dually infected patients was 0.46 (95% CI 0.37-0.57) and 0.80 (95% CI 0.61-1.05), respectively. Likewise, male gender, age of more than 30 years and single marital status were risk factors of LP. Fewer HIV-2 infected patients (21.7%) than HIV-1 infected patients (23.5%) were late presenters (aOR 0.57, p<0.01).

**Table 2:** Risk factors of late presenters with advanced disease (AD) versus non-late presenters (non-LP)

	Advanced disease (AD) <200 cells/µL			
	Univariable analysis		Multivariable analysis	
	OR (95% CI)	P-value	aOR (95% CI)	P-value
**HIV-type**				
HIV-1	1.00	-	1.00	-
HIV-2	0.49 (0.40-0.60)	<0.01	0.46 (0.37-0.57)	<0.01
HIV-1/2	0.82 (0.63-1.05)	0.12	0.80 (0.61-1.05)	0.11
**Sex**				
Female	1.00	-	1.00	-
Male	1.66 (1.41-1.96)	<0.01	1.49 (1.24-1.80)	<0.01
**Age stratified**				
Age ≤ 30 years	1.00	-	1.00	-
Age 30-49 years	1.52 (1.27-1.81)	<0.01	1.66 (1.36-2.02)	<0.01
Age ≥ 50 years	1.10 (0.85-1.42)	0.45	1.48 (1.10-1.99)	0.01
**Marital status**				
Married	1.00	-	1.00	-
Divorced	0.99 (0.72-1.36)	0.96	1.03 (0.74-1.44)	0.86
Widowed	0.79 (0.63-0.99)	0.04	1.00 (0.78-1.28)	0.98
Single	1.06 (0.88-1.29)	0.53	1.30 (1.05-1.61)	<0.01
**Ethnicity**				
Balanta	1.00	-	1.00	-
Fula	1.47 (1.14-1.89)	<0.01	1.55 (1.19-2.02)	<0.01
Mancanha	1.05 (0.74-1.51)	0.77	1.15 (0.79-1.68)	0.45
Mandinga	2.04 (1.47-2.83)	<0.01	2.10 (1.50-2.95)	<0.01
Manjaco	1.20 (0.87-1.67)	0.27	1.22 (0.87-1.72)	0.25
Pepel	1.29 (0.94-1.79)	0.12	1.28 (0.91-1.81)	0.16
Other^+^	1.29 (0.99-1.68)	0.06	1.35 (1.03-1.78)	0.03
**Attended school^++^**				
Yes	1.00	-	-	-
No	0.94 (0.80-1.11)	0.47	-	-
**Year of diagnosis**				
2007	1.00	-	1.00	-
2008	0.78 (0.52-1.15)	0.21	0.71 (0.47-1.09)	0.12
2009	0.68 (0.47-1.00)	0.05	0.64 (0.43-0.96)	0.03
2010	0.56 (0.39-0.82)	<0.01	0.51 (0.34-0.75)	<0.01
2011	0.50 (0.34-0.73)	<0.01	0.45 (0.30-0.68)	<0.01
2012	0.60 (0.40-0.90)	0.01	0.52 (0.34-0.79)	<0.01
2013	0.63 (0.42-0.95)	0.03	0.50 (0.33-0.78)	<0.01

+ Other ethnicities: Beafada, Bijago, Caboverdeano, Felupe, Mansonca and mixed

++ Not including koranic school

### Initiation of ART

ART was initiated among 2,395 (64.4%) patients during the study period, and this proportion was higher among patients with LP (76.8%) and AD (76.6%) than among patients with non-LP (33.2%), p < 0.001. The overall median time to ART initiation was 18 days (IQR: 9 - 49 days). The median time to ART initiation was lowest for patients with AD (14 days) followed by patients with LP (20 days) and non-LP (266 days, p < 0.001). Only 44 (1.2%) patients initiated ART the same day as they were diagnosed with HIV.

### Mortality of patients

**Figure 3:**
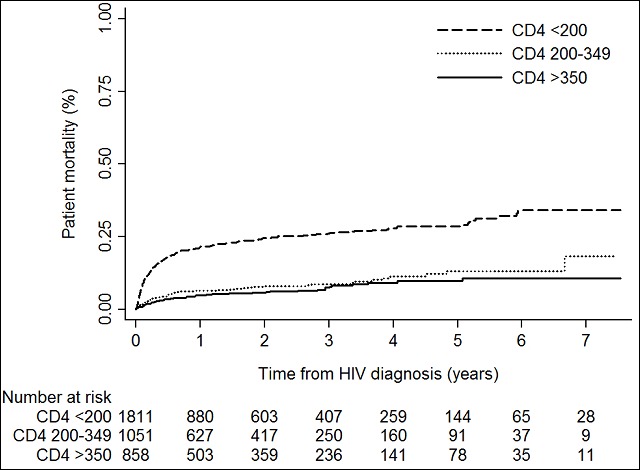
Patient mortality by time from HIV diagnosis

All patients were included in the mortality analysis and contributed with 6,950 person-years of observation. During follow-up, 532 deaths were registered; overall mortality rate (MR) = 7.7 (95% CI: 7.03 - 8.33) per 100 person-years. Two-hundred-and-nine (39.3%) of all deaths occurred before initiation of ART (Figure 3). The mortality was most apparent during the first year after HIV diagnosis where 64.3% of the deaths occurred. The mortality rate (MR) was higher among patients with AD (MR 13.1 per 100 person-years (95% CI: 11.9 - 14.4)) than LP (MR 3.8 per 100 person-years (95% CI: 3.0 - 4.8)) and non-LP (MR 2.9 per 100 person-years (95% CI: 2.3 - 3.8)). MRR for AD vs. non-LP was 3.82 (95% CI: 2.9 - 5.0, p < 0.001). There was no significant difference in the mortality between LP and non-LP; MRR 1.31 (95% CI 0.92 - 1.85, p=0.13)

### Patients lost to follow-up and patients transferred

According to our definition, 2,169 (58.3%) patients became lost to follow-up during the study period. The proportion of patients lost to follow-up was higher among non-late presenters (67.8%) and patients with LP (60.5%) compared with AD (51.8%, p < 0.001). In total, 178 (4.8%) patients were transferred to another facility with no difference between groups; non-LP (4.5%), LP (5.9%) and AD (4.4%, p=0.19). By the end of the study period 841 (22.6%) of the patients were alive on follow-up.

## Discussion

In this cohort, 65.7% of the HIV infected patients had a CD4 cell count measured within the first 90 days of the HIV diagnosis. Almost half the patients presented with AD and additionally one quarter were late presenters. Risk factors of AD were male sex, age > 30 years, marital status single, Fula and Mandinga ethnicity. HIV-2 and HIV-1/2 dually infected patients were less likely to be late presenters. Although ART was initiated for 64.4% of patients, those with AD presentation had an almost four times higher mortality. The Bissau HIV cohort is, to our knowledge, the largest single-center HIV cohort globally in terms of enrolled patients with HIV-2 and HIV-1/2 dual infection. Only few clinical studies on HIV-2 and HIV-1/2 infection have been performed. In sub-Saharan Africa HIV cohorts are characterized by a high rate of loss to follow-up [10, 19] and in a meta-analysis a combined 40% of patient lost to follow-up had in fact died. Thus loss to follow-up in our study may have underestimated the true mortality rate [20]. Another limitation is the uncertainty in the HIV typing. We have previously shown that the rapid test used in Guinea-Bissau for HIV type discrimination (SD Bioline HIV 1/2 3.0) may overestimate the number of HIV-1/2 dually infected patients [21, 22] and the successor (First Response HIV Card Test 1-2.0) has been shown to produce a high number of HIV-positive untypable results. Different definitions of LP have previously been used ranging from a CD4 cell count < 50 cells/µL [23] to CD4 cell count < 350 cells/µL [2]. Other studies have used the term “late diagnosis“ as reviewed in a European study [24] and some differentiate between LP and AD whereas others do not [17]. These differences challenge direct comparison between studies. A large study from 132 facilities in Kenya, Mozambique, Rwanda and Tanzania included 334,557 HIV infected adults of whom 19% had AD (CD4 cell count < 100 cells/µL). Risk factors of LP were tuberculosis treatment and a gap of = 12 months gap in pre-ART care [12]. In north-east Ethiopia, 160 cases of patients with CD4 cell count < 200 cells/µL or WHO clinical stage 3-4 (termed late presenters) were compared with 160 controls of patients with CD4 cell count ≥ 200 cells/µL or WHO clinical stage 1-2. The cases more often lived with their families or in a rented house, were non-pregnant women, perceived ART having many side effects, perceived HIV as a stigmatizing disease, tested with sickness/symptoms, did not disclose their HIV status to their partner, frequent alcohol use and had spent more than 120 months with their partner at HIV diagnosis [25]. Among 332 young women in central Mozambique late diagnosis (CD4 cell count < 350 cells/µL) was associated with lack of knowledge of the HIV status of the primary partner and having a gynecological pathology in the last year [26]. In South Africa, 33.6% of 830 patients presented late (CD4 cell count <100 cells/µL), which was associated with living far away from test site, working outside the home, perceiving health service barriers and/or having poor emotional health [27]. A total of 2,311 HIV infected patients were included in a study from Uganda and 40% were late presenters (WHO disease stage 3 or 4). Predictors of late presentation were age 46-60 years (vs. younger), lower educational level, being unemployed, living in a household with others, being unmarried and lack of spousal HIV status disclosure [28]. The numerous risk factors of LP/AD mentioned above indicates the diversity in explanatory causes of late HIV testing and demonstrates the need for local studies. Causes of LP/AD may differ with geographical region and local settings. As in our study, male sex was a risk factor of LP in many of these African studies possibly reflecting a poorer health-seeking behavior in the male population [12, 26–29]. In Bissau male sex is also associated with higher rates of loss to follow-up after CD4 cell count measurement [10].

### Implications

Among all patients diagnosed with HIV at our clinic, 33.1% did not have a CD4 cell count measured within 90 days of HIV diagnosis and were excluded from further analysis. Due to unstable supply of reagents for flow cytometry and because of machine breakdowns, CD4 cell counts were not available for patients several times during the study period. Furthermore, Guinea-Bissau has been considered politically unstable for many years and coup attempts have closed down HIV clinic whereas the laboratory have been closed down occasionally because of work-force strikes when salaries were not paid [30]. These mere practical issues may explain why excluded patients did not differ much from the patients with a CD4 cell count measured. Only 18.3% of the patients in this study had a CD4 cell count performed on the same day as the HIV diagnosis and 1.2% initiated ART on the day of the HIV diagnosis. In Bissau, the CD4 cell counts were measured at a central laboratory across town which meant, that the result would usually not be available for clinicians until at least the day after and physicians may have been reluctant to initiate ART before knowing the CD4 cell count of the patient. This would have caused delayed ART initiation with a risk of the patients not showing up at the clinic again; a missed opportunity. Point-of-care CD4 testing has been evaluated in a recent review analysis including 15 studies mainly from sub-Saharan Africa. When comparing with conventional laboratory-based testing, the point-of-care CD4 testing increased the likelihood of having a CD4 cell count measured and having received the CD4 result. Overall, time between HIV diagnosis and CD4 measurement was reduced by 9 days [31]. However, the performance of different point-of-care devices for CD4 cell count varies with regard to accuracy and costs [32]. The implementation of point-of-care monitoring tools at decentralized clinic laboratories may therefore be warranted [33]. HIV-2 infected patients presented with higher CD4 cell counts in our study. Individuals with HIV-2 infection often have slower disease progression compared with those with HIV-1 infection [14, 15]. A proportion of the patients enrolled under care at the HIV clinic at HNSM are tested for HIV infection as part of routine screenings procedures (pregnant women, students, blood donors) explaining their referral to the HIV clinic and early presentation. Controversially, studies have suggested that initial infection with HIV-2 may protect against subsequent HIV-1 disease progression [34, 35] whereas other studies did not find a protective effect [36]. Based on the HIV tests used, we found that HIV-1/2 dually infected patients were significantly less likely to have AD than HIV-1 infected patients. A recent meta-analysis of 56 studies from Sub-Saharan Africa found that the overall median CD4 cell count in 2002 was 251 cells/µL [37]. The median CD4 count was somewhat lower in our study (206 cells/µL). Due to intermittently unavailable CD4 cell count measurements and low stock or no HIV tests in Bissau, physicians may have prioritized blood analyses for the clinically sickest patients. The aforementioned meta-analysis also found that the CD4 cell count did not change between 2002 - 2013, except for patients living in South Africa. We observed an increase in CD4 cell count at presentation from 2007 to 2011. CD4 cell count measurements and large scale ART did not become available in Guinea-Bissau until 2007, and the first patients enrolled in care were likely those in greatest needs of care. The relatively late introduction of ART in Guinea-Bissau may also explain why age >30 years was a risk factor of AD in our study period. On average, time since infection may be longer for older patients causing more severe disease progression. Low self-perceived risk of HIV infection among older patients may also explain later presentation [28]. Cultural differences between different ethnicities may influence time of presentation at an HIV clinic. Fula and Mandinga ethnicity, which were associated with AD in this study, constitutes two major population group in West Africa. In Guinea-Bissau distinct living conditions and cultural patterns are practiced in these ethnic groups, participation in vaccination campaigns are lower and child mortality is higher than in other groups [38–41]. Similarly, the mortality rate was higher in these ethnic groups among women of reproductive age [42]. HIV-1 infection was also associated with Mandinga ethnicity in a study among pregnant women in Bissau [43]. Association between AD and Fula/Mandinga ethnicity may reflect differences in geographic origin [42], socioeconomic characteristics and health seeking behavior [40]. During the study period, the WHO recommendations for initiation of ART based on CD4 cell count changed. Previously only patients with CD4 cell count below 200 cells/µL were recommended to initiate ART, but in 2010 the recommendations changed to 350 cells/µL or less. The national guidelines in Guinea-Bissau have followed these recommendations from WHO [44], although the newest guidelines suggesting ART initiation at a CD4 cell count below 500 cells/µL had not yet been implemented at the end of the study period. ART is provided free-of-charge for patients, but still, approximately 25% of patients with AD in our study did not initiate ART. Early mortality was very high for patients with AD, but may be improved with rapid initiation of ART. We have previously described that loss to follow-up bears a significant cause of the failure to start ART both before and after the first CD4 cell count [10].

### Unanswered questions for future research

As patients with AD have higher mortality rates, it should be of high priority to investigate the causes of LP/AD at HIV testing. Additional HIV screening of the population among high-risk groups may induce infected individuals to be enrolled under care at an earlier point.

## Conclusion

A high proportion of HIV infected patients had AD and these patients exhibited a much higher mortality. Initiatives to enroll patients in care at an earlier point are needed and should focus on risk groups.

### What is known about this topic

- Late presentation of HIV infected patients causes delayed initiation of antiretroviral therapy;
- In other settings, late presentation has been associated with higher mortality and impaired CD4 cell count increment;
- Little is known about the extent and significance of late presentation in Guinea-Bissau.

### What this study adds

- More than half of the HIV infected patients in Bissau were late presenters;
- HIV-1 infected patients presented with lower CD4 cell count than HIV-2 infected patients;
- Dispite antiretroviral therapy, late presenters with advanced disease progression had an almost 4 times higher mortality rate.
